# Functional Nanostructures for Sensors, Optoelectronic Devices, and Drug Delivery

**DOI:** 10.3390/nano10061195

**Published:** 2020-06-19

**Authors:** Maria Angela Castriciano

**Affiliations:** Istituto Per Lo Studio Dei Materiali Nanostrutturati, c/o Dipartimento di Scienze Chimiche, Biologiche, Farmaceutich ed Ambientali, University of Messina, 31 98166 Messina, Italy; maria.castriciano@cnr.it

Nanoparticles and nanostructured materials represent an active area of research for their impact in many application fields. The recent progress obtained in the synthesis of nanomaterials and the fundamental understanding of their properties led to significant advances for their technological applications. The scope of the Special Issue “Functional Nanostructures for Sensors, Optoelectronic Devices, and Drug Delivery” was to provide an overview of the current research activities in the field of nanostructured materials, with a particular emphasis on their potential applications for sensors [1,2,3,4,5,6,7,8,9], optoelectronic devices [10,11,12,13,14,15], and biomedical systems [16,17,18]. The Special Issue welcomed the submission of original research articles [1,2,3,4,5,6,9,10,11,12,13,14,15,16,17] and comprehensive reviews that demonstrated or summarized significant advances in the above-mentioned research fields. Next, the Special Issue collected fifteen selected original research papers and three comprehensive reviews [7,8,18] on various topics of nanostructured materials and relative characterization spanning from fundamental research to technological applications. More than 100 scientists from universities and research institutions participated with their research activities and expertise in the success of this Special Issue.

The scientific contributions are summarized here.

A facile one-step hydrothermal synthesis reaction for obtaining flower-like CeO_2_-doped SnO_2_ nanostructures was reported. The composite system showed improved gas-sensing performances, possibly due to the formation of n-n heterojunctions between CeO_2_ and SnO_2_ and due to the presence of Ce^4+^/Ce^3+^species in SnO_2_ that facilitate the interaction of electrons [1].

An easy meta-ion mediated hydrothermal route was successfully used to prepare cubic, thorhombic, and discal Fe_2_O_3_ NPs with a uniform size and controllable structure that were coupled with graphene oxide (GO) nanosheets. The influence of Fe_2_O_3_/GO morphologies on electrochemical sensing performances was studied systematically. The synergistic effect from d-Fe_2_O_3_ NPs and GO nanosheets operated as sensing films for the simultaneous detection of dopamine and uric acid [2].

The potential to detect ethanol vapor in an open environment was reported for ZnO–WO_3_ composite nanorods, which exhibited high selectivity for this alcohol among the various target gases of NH_3_, H_2_, and NO_2_. These composite nanorods were obtained by a combination of hydrothermal growth, sputtering methodologies, and a thermal annealing procedure in a hydrogen-contained atmosphere to induce a microstructural modification of the material that allowed for the improvement of its sensing performance. The composite nanorods annealed at 400 °C exhibited a strong response of 16.2 at the gas concentration of 50 ppm, while the pristine ZnO–WO_3_ could only reach 7.3 at the identical gas concentration [3].

Reproducible gas sensing responses to NO_2_ and H_2_ and humidity at 150 °C with detection limits of 200 ppb and 5 ppm to NO_2_ and H_2_, respectively, were reported for well-packed and interconnected WS_2_ flakes with controlled and reproducible microstructure over large areas. The WS_2_ flakes were obtained by a reproducible and high-yield exfoliation process followed by drop casting the centrifuged suspension, leading to the deposition of thin films, thus representing a fast, simple, and scalable method, compatible with standard microelectronic fabrication techniques [4].

Uniform and small-size Cu_2_O/Ag nanocomposites showing electrochemical behavior and good electrocatalytic reduction performance towards H_2_O_2_ were successfully synthesized by an easy one-step procedure. The linear range of the Cu_2_O/Ag/glass carbon electrode was estimated to be 0.2–4000 μM with a sensitivity of 87.0 μA mM^−1^ cm^−2^ and a low detection limit of 0.2 μM. The anti-interference capability experiment indicated that the Cu_2_O/Ag nanocomposites have good selectivity toward H_2_O_2_. Furthermore, the H_2_O_2_ recovery test in the milk solution demonstrated the Cu_2_O/Ag/GCE (glassy carbon electrode) potential application in routine H_2_O_2_ analysis [5].

New voltammetric sensors based on combinations of gold nanoparticles and sulfur-substituted zinc phthalocyanines, AuNP^tOcBr^/ZnPc^RS^ and AuNP^tOcBr^/ZnPc^R-^S-ZnPc^R^, have been developed and used as electrochemical sensors for the detection of catechol. The electron transfer process, as well as the existence of synergistic effects between both components in the absence and presence of covalent links, has been analyzed, showing that the investigated samples enhance the electron transfer rate of the catechol reduction [6].

J-aggregates of 5,10,15,20-tetrakis-(4-sulfonatophenyl)-porphyrin (TPPS) porphyrin are interesting nanomaterials, since by depending on the experimental conditions and templating agents, they showed a variety of different morphologies and physical-chemical properties. Easy and convenient methods to obtain, under mild acidic conditions, novel nanohybrid assembly of porphyrin J-aggregates with Au_10_ cluster or nanoparticles, or superparamagnetic iron oxide nanoparticles (SPIONs) incorporated in micelles, have been reported [9,10]. In the first case, J-aggregates showed a chirality that is related to the configuration of the amino acid (d- and l-histidine) used in the metal clusters synthesis suggesting the important role of Au_10_ clusters as chiral seeds in the growth of the porphyrin aggregates. Furthermore, the growth of the metallic nanostructures with the codeposition of TPPS J-aggregates on the substrates has been exploited as a test system for the potential use of these nanoparticles in SERS applications. This supramolecular system tends to spontaneously cover glass substrates with a co-deposit of gold nanoclusters and porphyrin nanoaggregates showing Surface-Enhanced Raman scattering (SERS) [9]. In the second system, the proper choice of experimental conditions and mixing protocol allows one to control the kinetics of growth of TPPS J-aggregates, leading to supramolecular structures in which the porphyrin nanoassemblies are embedded into the magnetic micelles. By applying an external magnetic field, a high level of alignment of the nanohybrids into the film has been achieved [10].

Hydrothermal methodology was used also for obtaining ZnO nanorods arrays with high surface area and well-aligned crystallographic orientation for fundamental studies and in optoelectronic applications, including visible-light-emitting devices and display systems. These nanostructures show, upon argon/SF_6_ plasma treatment, enhancement of the PL intensity in the orange/red region of ZnO_2_-fold, compared to the ZnO sample without plasma treatment. Moreover, the presence of hydroxyl group at the surface, more oxygen in the ZnO lattice (*O_L_*), fluorine incorporation in terms of F–Zn and F–OH bonds, and passivation of the surface states as well as bulk defects have been reported [11].

Kumar at al. reported on the low temperature hydrothermal synthesis and structural and photoelectrical characterization of Sn_0.97_Zn_0.03_S_2_ nanoflakes. They demonstrated that these nanostructured materials show higher visible-light absorption and significant improvement in conductivity and sensitivity to illuminations, compared to pristine SnS_2._ Such an excellent performance of Sn_0.97_Zn_0.03_S_2_ nanoflakes was reported for potential application in optoelectronic devices [12].

The impact of the host material composition on the temperature-dependent luminescent properties of vanadium-doped nanocrystalline garnets was investigated by Kniec et al. It was demonstrated that the incorporation of Ga^3+^ ions into the Y_3_Al_5−*x*_Ga*_x_*O_12_:V structure enables modification of the emission color of the phosphor by the stabilization of the vanadium ions on the V^4+^ oxidation. The abilities of the Y_3_Al_5−*x*_Ga*_x_*O_12_:V nanocrystals to noncontact temperature sensing in terms of spectral response, maximal relative sensitivity, and operating temperature range, by the Ga^3+^ doping, was also reported [13].

n-n heterostructures based on graphene monolayer and SnSe_2_ quantum dots (QDs) showing good performance in the light absorption and the transportation of photocarriers were reported by Li et al. Uniformly distributed SnSe_2_ quantum dots were synthesized at room temperature by a facile and environment-friendly methodology. The graphene monolayer and SnSe_2_ quantum dots UV-detector showed fast photoresponse time of ~0.31 s, and its photoresponsivity was up to 7.5 × 10^6^ mA W^−1^, which are promising for optoelectronic applications [14].

Turek et al. reported on an aqueous two-phase extraction (ATPE), which allowed to differentiate among large diameter single-walled carbon nanotubes (CNTs) by electrical character. The introduction of hydration modulators (H_2_O_2_ and PEGme) significatively improved the resolution of the one-step system. Moreover, to isolate the separated CNTs from the matrices, the authors proposed a method based on precipitation and hydrolysis, which is easier than lengthy dialysis routines [15].

Blue, green, and red fluorescent nanoparticles composed of thermosensitive liposomes (TSLs) of phospholipid 1,2-dipalmitoyl-sn-glycero-3-phospho-rac-(1-glycerol) sodium salt, and three different conjugated polyfluorenes were investigated as fluorescent drug carriers for bioimaging applications. These systems showed stable fluorescence signals, good colloidal stability, spherical morphology, and ability to transport and control drug delivery. Preliminary experiments with mammalian cells showed the capability of the nanoparticles to mark and visualize cells in blue, green, and red colors, extending their applications as bioimaging probes [16].

“Three bullet” nanoconstructs based on mesoporous silica nanoparticles (MSNs) covalently integrating a nitric oxide (NO) photodonor (NOPD) and a singlet oxygen (^1^O_2_) photosensitizer (PS) and encapsulating the anticancer doxorubicin (DOX) in a noncovalent fashion were reported as good candidates for potential combined cancer photo-chemotherapy. Such a multifunctional nanoplatform is able to generate ^1^O_2_ and NO under selective excitation with green and blue light, respectively, and release the noncovalently entrapped anticancer DOX under physiological conditions. Preliminary biological results performed using A375 cancer cells showed a good tolerability of the functionalized MSNs in the dark and a potentiated activity of DOX upon irradiation, due to the effect of the NO photoreleased [17].

Beside the fascinating samples reported so far, the Special Issue includes interesting comprehensive reviews on nanostructured organic and hybrid compounds [7]; bare iron oxide nanoparticles (BIONs) [8]; and microbial biosynthesis of nanomaterials by bacteria, yeast, molds, and microalgae for the manufacturing of sensoristic devices and therapeutic/diagnostic applications [18]. In particular, Prosa et al. reported an interesting overview on the use of nanostructured organic and hybrid compounds in optoelectronic, electrochemical, and plasmonic components as constituting elements of miniaturized and easy-to-integrate biochemical sensors. They highlight that the new concept of having highly integrated architectures through a system-engineering approach may enable the full expression of the potential of the sensing systems in real-setting applications in terms of fast-response, high sensitivity, and multiplexity at low-cost and ease of portability [7]. Madro et al. highlighted the properties and advantages of BIONs with respect to pristine surface chemistry of iron oxide, providing ideas on the future expansion of these nanomaterials and emphasizing the opportunities achievable by tuning their pristine surfaces [8]. Biosynthesis of nanomaterials by microorganisms, attracting interest as a new, exciting approach, was herein reported. Grasso et al. reported on microbial nanotechnology as a fascinating and booming field for future breakthrough nanomaterial synthesis. The authors showed that, through a ‘‘green’’ and sustainable approach, microbial nanotechnology can really spur innovation in nanomanufacturing, with a potential strong impact in several fields, including sensoristics and biomedicine [18].

In conclusion, the papers collected in this Special Issue reflect the existing widespread interest in design, synthesis, characterization, and potential applications of functional nanostructured materials. Although the present Special Issue cannot fully reproduce the complete topic of functional nanostructured materials, I am confident that its contributions to fundamental research will open new perspectives in development and innovation, thus improving the application of these materials in technological fields.
